# Long-Standing Seborrheic Keratosis of the Scalp

**DOI:** 10.7759/cureus.96830

**Published:** 2025-11-14

**Authors:** Jean El Hajj, Claudia Chidiac, Myriam Hajj, Roland Tomb

**Affiliations:** 1 Dermatology, Hotel Dieu de France, Beirut, LBN; 2 Dermatology Department, Saint Joseph University, Beirut, LBN

**Keywords:** benign tumor, differential diagnosis, scalp, seborrheic keratosis, skin histopathology

## Abstract

Seborrheic keratosis (SK) is a common benign epidermal tumor that frequently arises in elderly patients. Although it is usually straightforward to diagnose, atypical long-standing or pigmented lesions on the scalp can mimic malignancies such as melanoma or pigmented basal cell carcinoma. We present the case of an 82-year-old man with a slowly enlarging verrucous plaque on the left temporo-parietal scalp that has been present for at least 20 years. The lesion was asymptomatic but demonstrated dark pigmentation and irregular borders, prompting histopathologic evaluation. Biopsy confirmed SK, and the lesion was completely removed by shave excision and curettage. This case highlights the diagnostic challenge posed by long-standing pigmented scalp lesions and emphasizes the importance of clinicopathologic correlation to exclude malignancy.

## Introduction

Seborrheic keratosis (SK) is among the most common benign epidermal proliferations in adults, arising from clonal expansion of basaloid keratinocytes [1]. These tumors typically appear as sharply demarcated, waxy, or verrucous plaques with a characteristic “stuck-on” appearance. Their prevalence increases with age, and lesions are most frequently found on sun-exposed areas such as the head and trunk. Dermoscopy generally facilitates the distinction of SK from malignant lesions, though its accuracy may be limited in thick, hyperkeratotic, or large lesions where classic features are obscured [2].

Despite their harmless nature, SKs can sometimes mimic malignancy when they display irregular pigmentation or rapid growth, particularly on chronically sun-exposed areas such as the scalp [3]. The diagnostic overlap with melanoma or pigmented basal cell carcinoma can lead to unnecessary concern or, conversely, to misdiagnosis if histologic confirmation is omitted [4]. Few reports describe long-standing scalp SKs with pseudo-malignant features. This case underscores the diagnostic pitfalls of long-standing pigmented scalp lesions and illustrates the need for histopathologic verification to ensure accurate diagnosis and appropriate management.

## Case presentation

An 82-year-old man presented to the dermatology department with a pigmented lesion of the left temporo-parietal scalp that had been slowly enlarging for approximately 20 years. According to the patient, the lesion had gradually darkened and thickened but remained asymptomatic, with no pain, bleeding, pruritus, ulceration, induration, or discharge. His medical history was unremarkable, with no prior cutaneous malignancies or trauma to the area.

**Figure 1:**
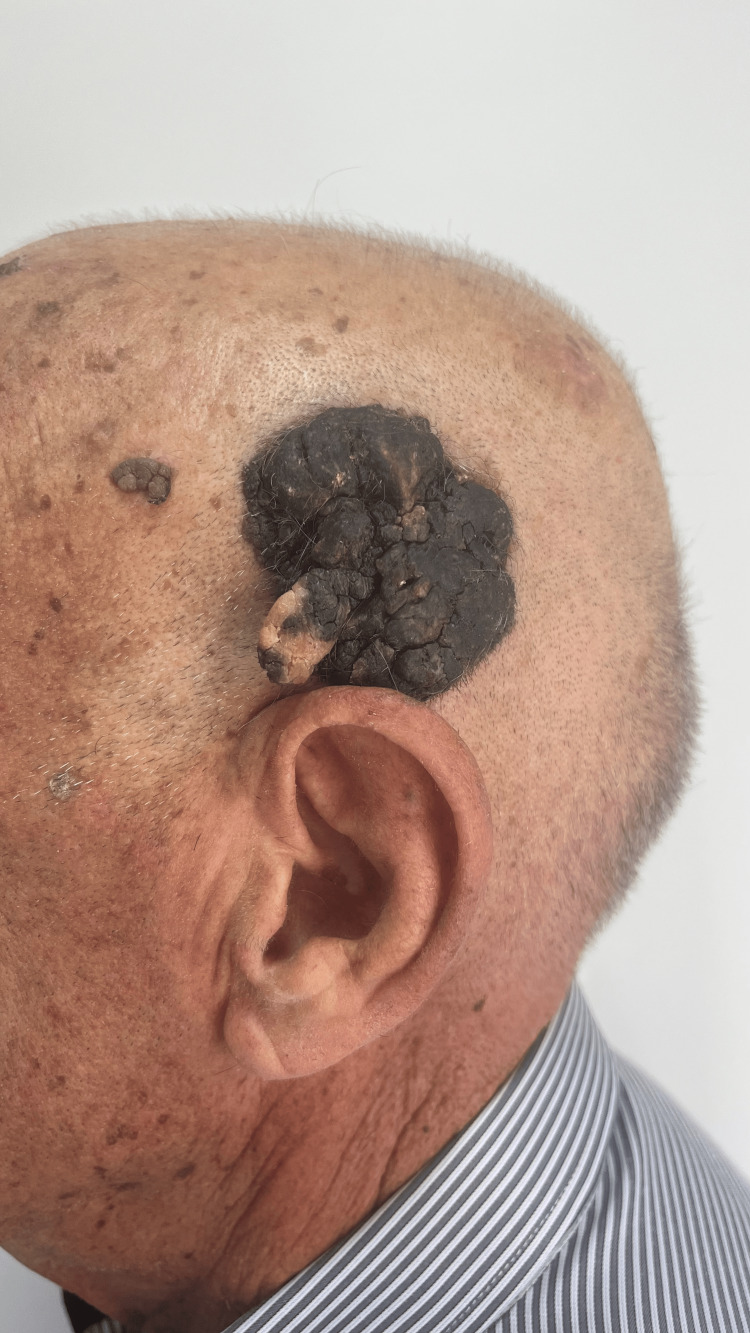
Large dark brown to black verrucous plaque with an uneven hyperkeratotic surface on the left temporo-parietal scalp.

Physical examination revealed a 5.0 × 6.5 cm oval, sharply demarcated verrucous plaque, dark brown to black in color, with an uneven hyperkeratotic and rough surface (Figure 1). The lesion appeared adherent but not ulcerated. Dermoscopy was limited by pronounced surface hyperkeratosis, revealing only heterogeneous yellow-brown keratotic areas without distinct structures. No cervical lymphadenopathy was detected.

**Figure 2:**
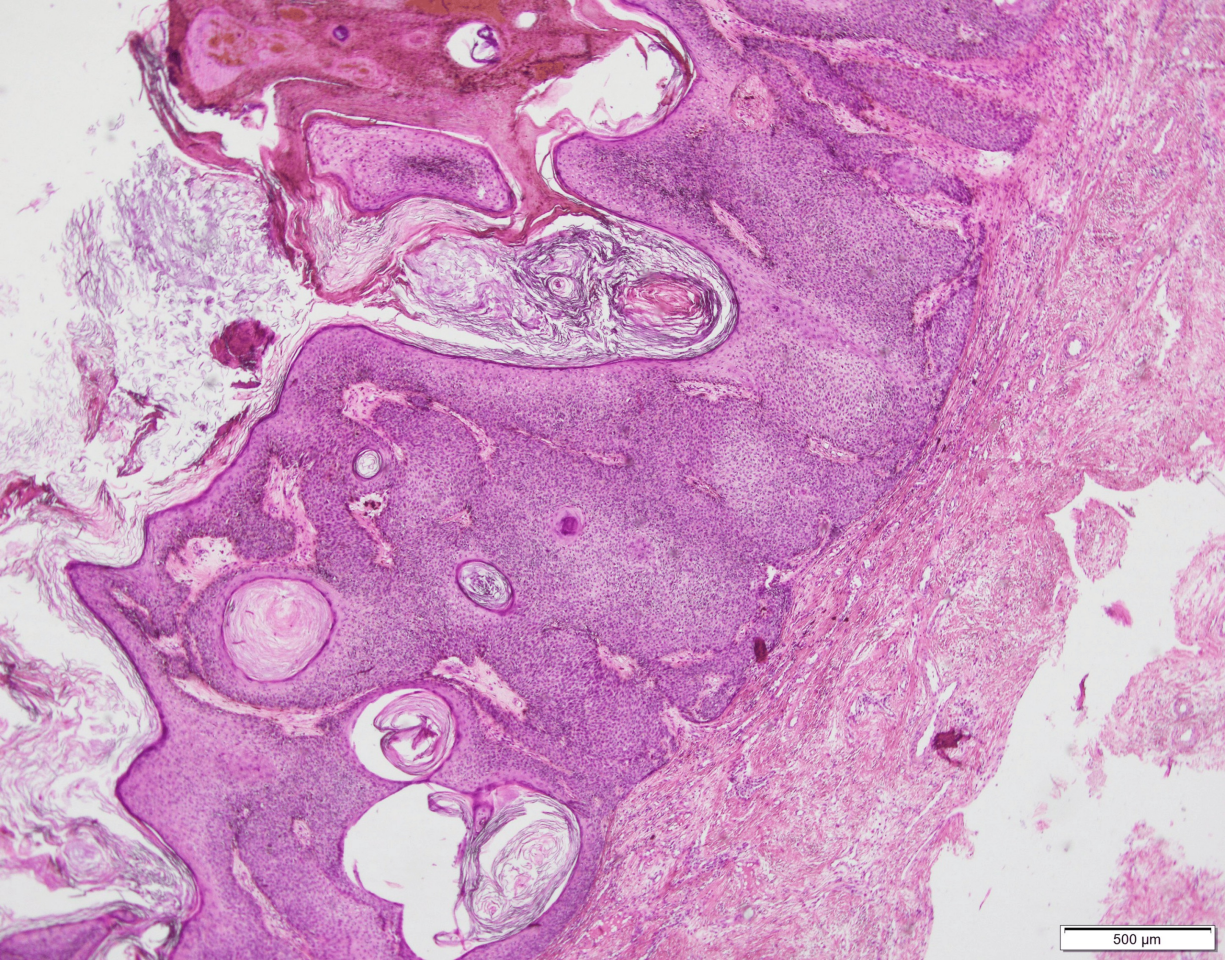
Histopathologic examination (H&E stain, ×100) showing characteristic features of seborrheic keratosis such as papillomatous epidermal hyperplasia with acanthosis, hyperkeratosis, and multiple horn cysts.

Differential diagnoses included seborrheic keratosis, epidermal nevus, verruca vulgaris, pigmented basal cell carcinoma, squamous cell carcinoma, and melanoma. Given the lesion’s chronicity and irregular pigmentation, an incisional biopsy was performed. Histopathologic analysis revealed papillomatous epidermal hyperplasia with acanthosis, hyperkeratosis, and multiple horn cysts. No cytologic atypia or melanocytic proliferation was observed, confirming the diagnosis of SK (Figure 2).

The lesion was completely removed by shave excision and gentle curettage under local anesthesia. The patient returned two weeks later for adjunctive cryotherapy to the curetted base. The wound healed by secondary intention without complication. No recurrence or new lesions were observed during 6 months of follow-up.

## Discussion

Seborrheic keratoses are benign epidermal tumors that increase in number and size with age [5]. Chronic ultraviolet exposure and genetic predisposition are considered key factors in their development [6]. Although SKs are classically described as flat, waxy, or verrucous growths with uniform pigmentation, long-standing lesions can evolve into thick, dark, and irregular plaques, especially on the scalp, where repeated trauma and photoaging occur.

In our patient, the lesion measured approximately 5.0 × 6.5 cm and had progressively darkened and thickened over two decades. Clinically, the differentiation between SK and malignant tumors such as melanoma can be challenging [7]. The “stuck-on” look of SK may help, but this is not pathognomonic. Dermoscopy was not helpful in this case because marked hyperkeratosis obscured the typical features of SK, such as milia-like cysts, comedo-like openings, and fissures [8].

Compared with typical SKs, which are generally smaller, uniformly pigmented, and found on the trunk or face, this lesion’s large size, dark pigmentation, and scalp localization made it an atypical presentation. Although the scalp is a recognized site for SK, it is less frequently affected than the trunk or face, with reported involvement in approximately 15-20% of cases, particularly among elderly males [9]. This may be related to chronic photoaging and repeated mechanical irritation. Taken together, the long duration, increased thickness, atypical pigmentation, scalp site, and lack of typical dermoscopic findings raised suspicion for malignancy and prompted a biopsy to rule out cutaneous cancers.

Histologically, SK is characterized by acanthosis, hyperkeratosis, and pseudohorn cysts [10]. The absence of melanocytic atypia distinguishes it from melanoma, the lack of basaloid palisading from basal cell carcinoma, and the absence of keratinocyte atypia or dermal invasion from squamous cell carcinoma [11]. In our case, the pathology findings were classic for hyperkeratotic SK, confirming a benign lesion despite its alarming clinical appearance. Similar long-standing scalp SKs are rarely reported in the literature, making this case a valuable addition to existing data.

From a therapeutic standpoint, treatment of SK is usually sought for cosmetic or symptomatic reasons. Common options include cryotherapy, shave excision, curettage, or electrodesiccation [12]. In this case, shave excision followed by gentle curettage was performed to achieve both complete removal and histopathologic confirmation while minimizing scarring. Adjunctive cryotherapy was then applied to the base to reduce the risk of recurrence. At the six-month follow-up, no recurrence or new lesions were observed.

The clinical message from this case is twofold. First, even lesions that have been stable for decades may acquire irregular pigmentation that mimics malignancy; therefore, duration alone should not preclude biopsy. Second, clinicians should maintain a high index of suspicion for skin cancers in elderly patients presenting with new or changing pigmented lesions, particularly on sun-exposed scalp areas. Correlating clinical, dermoscopic, and histopathologic findings ensures accurate diagnosis and appropriate management.

## Conclusions

This case demonstrates how long-standing seborrheic keratoses of the scalp may clinically resemble malignant pigmented lesions, particularly in elderly patients. Although benign, such lesions warrant biopsy when they exhibit irregular pigmentation, rapid growth, or atypical features. Histopathologic examination remains the gold standard for definitive diagnosis. Early recognition and confirmation can prevent misdiagnosis, unnecessary anxiety, and overtreatment.

## References

[1] Hafner C, Vogt T (2008). Seborrheic keratosis. J Dtsch Dermatol Ges.

[2] Moscarella E, Brancaccio G, Briatico G, Ronchi A, Piana S, Argenziano G (2021). Differential diagnosis and management on seborrheic keratosis in elderly patients. Clin Cosmet Investig Dermatol.

[3] Kerouach A Jr, Hali F Sr, Belanouane S, Marnissi F, Chiheb S (2022). Verrucous melanoma of the scalp initially misdiagnosed as seborrheic keratosis. Cureus.

[4] Zabel RJ, Vinson RP, McCollough ML (2000). Malignant melanoma arising in a seborrheic keratosis. J Am Acad Dermatol.

[5] Yeatman JM, Kilkenny M, Marks R (1997). The prevalence of seborrheic keratoses in an Australian population: Does exposure to sunlight play a part in their frequency?. Br J Dermatol.

[6] Heidenreich B, Denisova E, Rachakonda S (2017). Genetic alterations in seborrheic keratoses. Oncotarget.

[7] Braun RP, Rabinovitz HS, Krischer J (2002). Dermoscopy of pigmented seborrheic keratosis: A morphological study. Arch Dermatol.

[8] Carrera C, Segura S, Aguilera P (2017). Dermoscopic clues for diagnosing melanomas that resemble seborrheic keratosis. JAMA Dermatol.

[9] Roh NK, Hahn HJ, Lee YW, Choe YB, Ahn KJ (2016). Clinical and histopathological investigation of seborrheic keratosis. Ann Dermatol.

[10] Phulari RG, Buddhdev K, Rathore R, Patel S (2014). Seborrheic keratosis. J Oral Maxillofac Pathol.

[11] Luo W, Liang Y, Yang X, Wu W, Lu J (2025). Histopathological subtypes and clinical presentation of seborrheic keratosis: A 15-Year retrospective analysis of 1,169 cases in Hainan, China. Clin Cosmet Investig Dermatol.

[12] Gorai S, Ahmad S, Raza SS (2022). Update of pathophysiology and treatment options of seborrheic keratosis. Dermatol Ther.

